# Pharmacy in Baltimore

**Published:** 1856-04

**Authors:** 


*Pharmacy in Baltimore.*—A correspondent informs us that the pharmaceutists of Baltimore have held a meeting preparatory to revivifying the "Maryland College of Pharmacy,"

(which for several years has ceased to exert any influence in that city,) or to establishing a new institution more likely to grow into usefulness. This is good news. Baltimore possesses some able pharmaceutists, who will give character to any movement in which they may take part, and it is greatly to be desired that all such should be enlisted, and every other well disposed member of the profession who is favorable to progress, notwithstanding such may be deficient themselves. We have always believed that the movement of 1842 was too exclusive, too few were embraced in it to render it popular, and, as a consequence, it did not succeed. The object of such organizations should be to raise the status of the entire body; its fruits should not be for those already enlightened, so much as for those who desire improvement; and the prominent part taken by the more educated should be viewed, as it generally deserves to be, as their good will offering. We are far from advocating an indiscriminate admission, but every one, however small his pretensions, who has correct views of the vocation of the apothecary, should be included. We trust that the new organization will be in full operation by September next, when the American Pharmaceutical Association will meet in Baltimore, and claim fellowship through their regularly appointed delegates.—*Amer. Journ. of Pharm.*

